# Genomic signatures of climate adaptation in bank voles

**DOI:** 10.1002/ece3.10886

**Published:** 2024-03-07

**Authors:** Remco Folkertsma, Nathalie Charbonnel, Heikki Henttonen, Marta Heroldová, Otso Huitu, Petr Kotlík, Emiliano Manzo, Johanna L. A. Paijmans, Olivier Plantard, Attila D. Sándor, Michael Hofreiter, Jana A. Eccard

**Affiliations:** ^1^ Evolutionary Adaptive Genomics, Institute for Biochemistry and Biology, Faculty of Science University of Potsdam Potsdam Germany; ^2^ Comparative Cognition Unit, Messerli Research Institute University of Veterinary Medicine Vienna Vienna Austria; ^3^ CBGP, INRAE, CIRAD, Institut Agro, IRD Univ Montpellier Montpellier France; ^4^ Natural Resources Institute Finland Helsinki Finland; ^5^ Department of Forest Ecology, FFWT Mendel University in Brno Brno Czech Republic; ^6^ Laboratory of Molecular Ecology, Institute of Animal Physiology and Genetics Czech Academy of Sciences Liběchov Czech Republic; ^7^ Fondazione Ethoikos, Convento dell'Osservanza Radicondoli Italy; ^8^ INRAE, Oniris, BIOEPAR Nantes France; ^9^ HUN‐REN Climate Change: New Blood‐Sucking Parasites and Vector‐Borne Pathogens Research Group Budapest Hungary; ^10^ Department of Parasitology and Zoology University of Veterinary Medicine Budapest Hungary; ^11^ Department of Parasitology and Parasitic Diseases University of Agricultural Sciences and Veterinary Medicine Cluj‐Napoca Romania; ^12^ Animal Ecology, Institute for Biochemistry and Biology, Faculty of Science Berlin‐Brandenburg Institute for Biodiversity Research University of Potsdam Potsdam Germany; ^13^ Present address: Evolutionary Ecology Group, Department of Zoology University of Cambridge Cambridge UK

**Keywords:** *Clethrionomys glareolus*, climate gradient, genomic analysis, local adaptations, rodent

## Abstract

Evidence for divergent selection and adaptive variation across the landscape can provide insight into a species' ability to adapt to different environments. However, despite recent advances in genomics, it remains difficult to detect the footprints of climate‐mediated selection in natural populations. Here, we analysed ddRAD sequencing data (21,892 SNPs) in conjunction with geographic climate variation to search for signatures of adaptive differentiation in twelve populations of the bank vole (*Clethrionomys glareolus*) distributed across Europe. To identify the loci subject to selection associated with climate variation, we applied multiple genotype‐environment association methods, two univariate and one multivariate, and controlled for the effect of population structure. In total, we identified 213 candidate loci for adaptation, 74 of which were located within genes. In particular, we identified signatures of selection in candidate genes with functions related to lipid metabolism and the immune system. Using the results of redundancy analysis, we demonstrated that population history and climate have joint effects on the genetic variation in the pan‐European metapopulation. Furthermore, by examining only candidate loci, we found that annual mean temperature is an important factor shaping adaptive genetic variation in the bank vole. By combining landscape genomic approaches, our study sheds light on genome‐wide adaptive differentiation and the spatial distribution of variants underlying adaptive variation influenced by local climate in bank voles.

## Graphical abstract

We investigated adaptive differences in 12 bank vole populations across Europe in conjunction with geographic climate variation. We used multiple genotype‐environment associations, found that mean annual temperature was a shaping factor, and identified signatures of selection in candidate genes related to lipid metabolism and the immune system. Local climate is thus influencing adaptive variation in mammals.

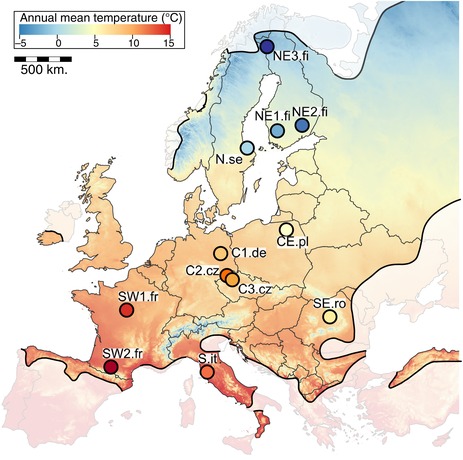

## 1 INTRODUCTION

Understanding how organisms adapt to their local environment is one of the central questions of evolutionary biology, which is becoming increasingly important in a world of human‐induced rapid climate change and environmental change. It is generally accepted that genetic variation within and among populations is influenced by the local environment in which organisms reside. For example, populations along an environmental gradient may be adapted to their local conditions if selection is strong enough relative to drift and gene flow between populations (Kawecki & Ebert, 2004). Since local adaptation arises from natural selection on adaptive phenotypic traits, it can be demonstrated by genetic differentiation at the genetic loci underlying those traits (Phifer‐Rixey et al., 2018; Stillwell, 2010; Stinchcombe et al., 2004). The genetic basis for environmental adaptation has been uncovered for a few obvious traits with distinct phenotypic characteristics, such as variation in coat colour in mice in relation to environmental background colour (Linnen et al., 2009; Nachman et al., 2003), reduction in armour plating in sticklebacks in response to freshwater colonization (Colosimo et al., 2005; Cresko et al., 2004), or body size and blood chemistry of house mice along a latitudinal cline in Eastern North America (Phifer‐Rixey et al., 2018). Rather than identifying specific phenotypic traits, signals of local adaptation can also be detected by scanning the genome for correlations between allele frequencies and environmental factors of interest after correcting for population structure. Such genetic‐environment association (GEA) tests are able to account for genome‐wide patterns caused by neutral processes such as gene flow and genetic drift (Coop et al., 2010; Frichot & François, 2015; Günther & Coop, 2013; Joost et al., 2007). The performance of GEA methods varies considerably depending on the sampling design, demographic history, and the amount of collinearity between neutral axes of population structure and environmental variables (de Villemereuil et al., 2014; Frichot et al., 2015; Whitlock & Lotterhos, 2015).

Signals of local adaptation are expected to affect only a small part of the genome, and most of the genome‐wide patterns at genetic loci are thought to come about by neutral processes, such as genetic drift, gene flow, or demographic history. When gene flow is reduced with increasing geographic distance because of limited dispersal, this results in a classic pattern of isolation by distance (IBD; Wright, 1943). Alternatively, isolation by environment (IBE) describes a pattern in which genetic differentiation increases with environmental differences independent of geographic distance. This might occur because of selection acting against maladapted immigrants or via other non‐adaptive processes affecting gene flow among populations in ecologically distant habitats (Sexton et al., 2014; Shafer & Wolf, 2013; Wang & Bradburst, 2014). Separating the effects of IBE from those of IBD can be challenging, as both patterns can result in similar patterns of genetic variation, for instance, when geographic distance is correlated with environmental distance (Bradburd et al., 2013; Meirmans, 2012), which is often the case in natural populations. Disentangling these effects can inform us about the relative contribution of both processes to patterns of genetic differentiation.

Theoretical and empirical studies suggest that many adaptive processes have a polygenic basis and are controlled by many genes of small effect (Barghi et al., 2020; Yeaman, 2015). Conventional univariate GEA methods aim at detecting alleles that correlate with (composite) environmental variables and are putatively under selection, focusing on only one locus at a time. Such methods are good at detecting signals from adaptive loci with large effects, but their ability to detect weaker signals of polygenic selection acting across many loci is rather limited (Rellstab et al., 2015; Wellenreuther & Hansson, 2016). In contrast, multivariate GEA methods, such as redundancy analysis (RDA), are able to take into account all environmental variation at the same time and can at the same time detect correlations between different sets of loci and different sets of environmental variables (Forester et al., 2018). By focusing on multiple loci at the same time, RDA is better at detecting weak multilocus signals of selection compared to univariate approaches (Capblancq et al., 2018; Forester et al., 2018), but it has not yet been widely used in evolutionary ecology. In addition, these multivariate approaches also allow the quantification of spatial patterns of adaptive genetic variation associated with environmental variables (Lasky et al., 2012; Micheletti et al., 2018; Nadeau et al., 2016).

Small forest mammals provide an ideal biological model to investigate the relative roles of selective and neutral factors in response to clinal environmental gradients because they have large geographic ranges and individuals are not highly mobile within the range (Haasl & Payseur, 2016). Such gradients and their respective genetic responses include climate and divergence in gene regulatory regions and genes related to metabolism and immunity (Phifer‐Rixey et al., 2018) or body size and extremities ratio (Ballinger & Nachman, 2022), rural‐urban gradients and signals of selection in genes involved in lipids and carbohydrates metabolism (Harris & Munshi‐South, 2017), as well as altitudinal gradients and genes related to metabolic function and oxygen transport (Beckman et al., 2022; Waterhouse et al., 2018).

**FIGURE 1:**
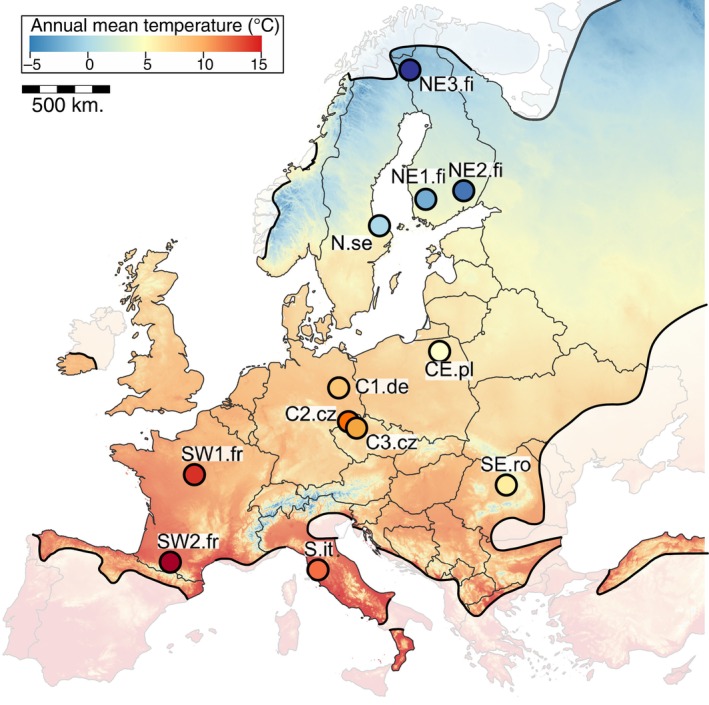
Sampling locations of populations (coloured circles) of *C. glareolus* in Europe with annual mean temperature [data: www.worldclim.org (Fick & Hijmans, 2017) and current distribution (Shenbrot & Krasnov, 2005)]. C, Central; E, Eastern; N, North; S, South; W, Western; .fi, Finland; .se, Sweden; .pl, Poland; .cz, Czechia; .de, Germany; it, Italy; .fr, France; .ro, Romania.

The bank vole *Clethrionomys glareolus* (also known as *Myodes glareolus*; Kryštufek et al., 2020) is a small Eurasian forest‐dwelling rodent with a broad geographic distribution in Europe, ranging from the Mediterranean peninsulas and the southern coast of the Black Sea in the south almost to the northern edge of Scandinavia (Figure 1). This distribution covers a wide temperature gradient (Figure 1). Bank voles survived in several refugia during the Last Glacial Maximum, including the well‐known refugia on the Mediterranean peninsulas and cryptic refugia in the Carpathians (Kotlik et al., 2006; Wójcik et al., 2010) and the Ural Mountains (Abramson et al., 2009; Deffontaine et al., 2005). Their subsequent recolonization of Europe, when the climate became more favourable at the beginning of the Holocene, resulted in a complex genetic structure with several distinct phylogeographic lineages, first described based on mitochondrial DNA sequences (Filipi et al., 2015) and later confirmed by genome‐wide SNP analyses (Horniková et al., 2021; Marková et al., 2020). Bank voles have limited dispersal capabilities (Deter et al., 2008; Viitala et al., 1994) and short generation times, resulting in large local effective population sizes. Together, these factors result in a large evolutionary potential for genetic responses to local conditions. Therefore, bank voles are a suitable system to study the signatures of local adaptation in response to spatially varying climate‐induced selective pressures along an environmental gradient. They have been the target of GEA studies in relation to geographic expansion (White et al., 2013) and tolerance to *Puumala orthohantavirus* infection (Rohfritsch et al., 2018). However, the specific selection forces driving their adaptations along wide latitudinal gradients, as well as the genetic loci involved, are not well understood.

The aim of this study is to investigate how population history and adaptation to local climate affect the spatial distribution of genomic variation in bank vole populations across Europe. We identified candidate genes and climate variables responsible for adaptive variation by testing for associations between allele frequencies and environmental variables using multiple univariate and multivariate GEA methods. We estimated the relative role of population structure versus environmental selection in explaining observed genetic variation by accounting for the neutral genetic structure.

## 2 METHODS

### 2.1 Vole samples and climate variables

Tissue samples of a total of 275 voles representing 12 widely dispersed populations (Figure 1) with 21–24 individuals per population (Table S1) were collected by the authors, covering a distance of 3200 km from the northeast in Finland (25.9° E 68.0° N) at the northern distribution limit to the southwest in France (0.8° E 43.2° N) and 2700 km from the southwest (France) to the southeast in Romania (25.1° E 46.6° N). For each population, values of 10 bioclimatic variables were downloaded from the WorldClim V2 dataset (Fick & Hijmans, 2017). Climate at the sampling sites ranged from −2 to 12.5°C for mean annual temperature, from 500 to 1000 (SD*100) for temperature seasonality, from 5.5 to 9.8°C for mean diurnal temperature range, from 480 to 1080 mm for mean annual precipitation, and from 11% to 50% for precipitation seasonality. To account for correlation among climate variables (Table S2), principal component analysis (PCA) was used to reduce dimensionality in R v3.4.4 using the prcomp function (R Core Team, 2018) (Figure S1). This resulted in two climate‐based principal components that together explained 80% of the total variation. PC1 explained 62.5% of the variation and was correlated with increasing temperature and precipitation variables (except for a negative correlation with temperature seasonality and precipitation seasonality), while PC2 explained 17.1% of the variation and was mainly correlated with an increase in precipitation variables and weakly correlated with decreasing temperature variables (Table S3).

### 2.2 Molecular methods

DNA was extracted using the DNEasy Blood & Tissue kit (Qiagen, Hilden, Germany), quantified using a Qubit 2.0 fluorometer (Life Technologies Inc., ON, Canada), and subjected to a double digest restriction‐associated DNA (ddRAD) sequencing protocol (Peterson et al., 2012, Supplement M1). Samples were grouped into pools of 48 individuals and cleaned with Speedbeads. Each pool was size‐selected for fragments of 300–400 bp in length using a Pippin Prep system (Sage Science, Beverly, MA, USA). This range should yield approximately 38,000 ddRAD loci, based on in silico digestion of the *C. glareolus* genome sequence (GCA_001305785.1) using SimRAD (Lepais & Weir, 2014). For each pool, we performed qPCR to determine the optimal number of PCR cycles based on the onset of the saturation phase on amplification plots (range: 11–14 cycles; Gansauge & Meyer, 2013). Pools were then amplified in four parallel reactions of 40 μL with primers that amplify only fragments containing both P1 and P2 adapters. The resulting libraries were sequenced in two separate runs on an Illumina NextSeq 500 with mid‐output kits. We first sequenced the libraries with 75 bp paired‐end (PE) and then performed another sequencing run with 150 bp PE sequencing.

### 2.3 Mapping and SNP calling

Cutadapt v1.4 (Martin, 2011) was used to remove adapter sequences and trim bases with a Phred score of less than 20 at the 3′ end, retaining only reads with a length greater than or equal to 35 bp. These were then demultiplexed and assigned to individuals using iPyrad v0.7.13 (Eaton, 2014). To increase mapping success, we used the published *M. glareolus* reference genome, which we improved with the pipeline Cross‐Species Scaffolding (Grau et al., 2018) using the prairie vole (*Microtus ochrogaster*) reference genome (GCA_000317375.1, Table S4). We considered only biallelic SNPs with a minimum base quality of 20, a minor allele frequency greater than 0.05, a minimum *p*‐value threshold for calling a SNP of 10^−6^, a minimum read depth of 5, and a maximum read depth of 100 per sample. In addition, a site had to be present in at least 12 individuals in each of the 12 populations to be considered.

### 2.4 Genetic diversity and population structure

We estimated diversity statistics within populations using ANGSD (Korneliussen et al., 2013). First, we calculated nucleotide diversity as the average number of pairwise differences (π) (Nei & Li, 1979) and as the proportion of segregating sites (θ_W_) (Watterson, 1975). We estimated genome‐wide heterozygosity as the proportion of heterozygous genotypes in the total number of genotypes for each individual based on its site frequency spectrum (SFS) and estimated the inbreeding coefficients (F) using ngsF (Vieira et al., 2013).

To assess population structure using PCA, we created a covariance matrix among individuals using ngsCovar from the ngsTools suite (Fumagalli et al., 2014) and calculated principal components in R v3.4.4 (R Core Team, 2018) using the ‘eigen’ function. The number of principal components explaining most of the population structure was determined from the scree plot of PCA (Cattell, 1966).

We assessed admixture among populations using NGSadmix (Skotte et al., 2013) with a number of clusters *K* ranging from 2 to 14. We repeated each analysis 20 times and reported the results of the highest likelihood analysis for each *K*. Finally, we calculated the pairwise *F*_ST_ in ANGSD using the shared SFS for each pair of populations. The results were also used to test for IBD by calculating the correlation between pairwise linearized *F*_ST_ values [*F*_ST_/(1 − *F*_ST_)] and log‐transformed pairwise geographic distance (Rousset, 1997). We subsequently tested for patterns of IBE, while controlling for IBD using partial Mantel tests. Environmental distances for the two climate‐based principal components and the climatic variables were computed as the Euclidean distance between pairs of populations using the *vegdist* function of the Vegan package (version 2.5‐4, Dixon, 2003) in R. Then the correlation between linearized *F*_ST_ and environmental distance was tested for each variable separately, while including geographic distance included a control variable. The significance of the *r* statistics for IBD and IBE tests was tested using 1000 permutation. To correct for multiple testing, a Benjamini and Hochberg FDR correction of 5% was applied (Benjamini & Hochberg, 1995).

### 2.5 Identification of loci associated with climate variation

To identify loci subject to climate‐induced selection, we searched for genomic markers that showed the strongest association between allele frequencies within populations and climatic conditions while controlling for the effect of population structure. We used the univariate approaches Bayenv2 (Coop et al., 2010) and LFMM (Frichot et al., 2013), together with a multivariate RDA. RDA allows the analysis of multiple environmental variables and covarying selection signals across a set of loci and facilitates the detection of adaptive processes that result in weak, multilocus signatures of selection (Forester et al., 2018). It was performed using the package ‘Vegan 2.5‐4’ in R (Dixon, 2003). As a conservative approach, we only considered loci as candidate loci when they were detected by at least two methods (Supplement M1, de Villemereuil et al., 2014).

### 2.6 Partitioning genetic variation between population structure and climate

We also used a series of RDAs to evaluate the amount of putatively neutral and adaptive genetic variation that could be attributed to population structure, climate, or their joint effects. For this purpose, we first performed separate (p)RDAs, including the population‐specific allele frequencies of the 20,500 neutral SNPs (excluding all outlier loci as detected by GEA methods) as a dependent matrix, to asses drivers of genome‐wide variation. Secondly, we assessed the drivers of adaptive variation at loci with signals of selection. For this, we performed additional (p)RDAs where we included the allele frequencies of subsets of outlier loci detected using different GEA methods (LFMM, Bayenv2, and RDA) as well as the subset of candidate loci as dependent matrices. We included two independent matrices representing climate (containing the climate variables that are also used to identify outliers) and population structure (containing the first four components of the ngsCovar analysis described above). We used RDA to estimate the amount of genetic variation exclusively explained by either population structure or climate by conditioning the effect of each independent matrix on the other. To further estimate which proportion of explained genetic variation was attributable to the joint effect of population structure and climate, we subtracted their exclusive effects from the total amount of genetic variation explained. This joint effect represents the shared effects of population structure and climate.

In addition to this, to identify climate variables that contributed most to adaptive variation, we performed RDAs on the subsets of outlier loci. Here, we assessed the amount of genetic variation explained by each climate variable by conditioning the effect of the respective climate variable on all other climate variables and population structure. The significance of each RDA was tested using an ANOVA performed with 1000 permutations. Finally, we identified the climate variable that was most strongly associated with variation in each outlier locus. For this, we extracted loci scores from the separate RDAs for each climate variable, and we normalized these scores to zero mean and unit variance. We then considered the climate variable with the highest absolute score as the one with the strongest influence on that locus.

### 2.7 Cumulative adaptive variation

We calculated a polygenic score for each individual to assess the cumulative adaptive genetic contribution of candidate outlier loci associated with each climate variable (Babin et al., 2017; Gagnaire & Gaggiotti, 2016). At the meta‐population level, we first identified the relationship between allele frequency and each climate variable for each candidate adaptive locus. For each individual, we then generated a score for each SNP by using the genotypes (0, 1, or 2). If the relationship was negative, we inverted the scores (to 2, 1, or 0) to obtain a positive relationship. Polygenic scores were then calculated at the individual level by summing the score of each SNP for a given climate variable, resulting in a separate individual polygenic score for each climate variable. To assess how well the cumulative signal of putatively adaptive alleles correlates with each climate variable, we then tested the correlation between individual polygenic scores and each variable using both a linear and a quadratic model, selecting the model with the lowest Akaike information criterion value as the best fit. To be able to compare between the GEA methods, we also performed the analysis on different subsets of outlier loci. We separately calculated polygenic scores for the outlier loci detected by RDA with and without correction for population structure in order to examine the effects of population structure on RDA results.

### 2.8 SNP annotation and gene ontology

We used the LastZ pairwise alignment tool v1.04.00 (Harris, 2007) to find homologous *M. ochrogaster* positions and thereby obtain functional annotations for candidate loci. We used a 20,000‐bp scaffold surrounding each candidate locus as a query in LastZ and the default options for calculating pairwise alignments. Only alignments with a bit score greater than 1000 and a query coverage of at least 50% were considered. If multiple alignments passed this filter, the alignment with the longest length and highest bit score was selected as the best match. If the loci were in protein‐coding regions, we used the UniProt database to examine gene function and find gene ontology (GO) terms. We performed an enrichment analysis using topGO (Alexa & Rahnenführer, 2010) in the “biological processes” category. We compared our list of candidate genes with all the genes in our dataset. We used Fisher's exact test and the *elim* algorithm to account for correlation in the topology of the GO graph, and reported the GO terms with a *p*‐value <.01 and at least four associated genes.

## 3 RESULTS

### 3.1 SNP dataset

We obtained 592.4 million reads from the two runs of sequencing. After filtering for low‐quality reads and assigning individuals to barcodes, on average, 1.62 Mio reads per individual aligned to our improved reference genome, with per‐population averages ranging between 0.96 Mio (Site NE1) and 2.61 Mio (C1) reads per population. Across all individuals, high‐quality reads covered an average of 17.99 Mio nucleotides of the genome (~0.71%). Average read depth across sites for each individual ranged from a minimum of 3.1 to a maximum of 15.5, with an average of 8.6 per individual. Using this data, a total of 21,892 SNPs passed filtering and were used in the genotype‐environment analyses. The dataset used to examine population structure consisted of 2476 variable sites with data for all individuals.

### 3.2 Genetic diversity within populations

The proportion of segregating sites (θ_W_) ranged between 1.6‰ (Site N.se, Figure 1) and 4.8‰ (SE.ro) with an average of 2.9‰ (Table 1). The average number of pairwise differences (π) ranged between 1.9‰ (N.fi) and 4.1‰ (S.it), with an average of 3.0‰. Observed heterozygosity across populations ranged from 1.2‰ (N.fi) to 2.5‰ (SE.ro). None of the genetic diversity measurements showed any clear spatial pattern. Inbreeding coefficients (*F*_IS_) were overall low.

**TABLE 1:** Overview of genetic diversity statistics (mean (standard deviation)) for each of the 12 sampled *Clethrionomys glareolus* populations.

Symbol	Site	Country	*n*	θ_W_ (10^−3^)	π (10^−3^)	Tajima's *D*	Heterozygosity (10^−3^)	*F*_IS_
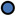	NE3	Finland (.fi)	24	2.6 (1.6)	2.9 (2.0)	0.33 (1.06)	1.9 (0.07)	0.014 (0.030)
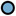	NE2	Finland (.fi)	24	2.5 (1.5)	2.8 (2.1)	0.29 (1.05)	1.8 (0.06)	0.004 (0.011)
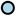	NE1	Finland (.fi)	23	2.4 (1.5)	2.6 (2.1)	0.24 (1.08)	1.6 (0.13)	0.007 (0.014)
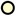	N	Sweden (.se)	24	1.6 (1.2)	1.9 (1.8)	0.32 (1.15)	1.2 (0.10)	0.003 (0.007)
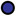	CE	Poland (.pl)	22	2.8 (1.5)	3.2 (2.0)	0.45 (0.93)	2.1 (0.03)	0.016 (0.025)
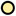	SE	Romania (.ro)	23	4.8 (2.1)	4.0 (2.3)	−0.67 (0.76)	2.5 (0.16)	0.028 (0.023)
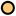	C1	Germany (.de)	24	2.6 (1.4)	3.1 (1.9)	0.57 (0.94)	2.0 (0.07)	0.025 (0.034)
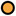	C2	Czechia (.cz)	24	3.5 (1.9)	3.4 (2.3)	−0.19 (0.90)	2.1 (0.15)	0.012 (0.026)
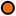	C3	Czechia (.cz)	23	3.3 (1.7)	3.4 (2.1)	−0.01 (0.88)	2.1 (0.08)	0.018 (0.025)
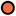	S	Italy (.it)	22	3.5 (1.8)	4.1 (2.4)	0.52 (0.85)	2.5 (0.16)	0.012 (0.029)
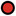	SW1	France (.fr)	21	2.9 (1.4)	3.3 (2.0)	0.44 (0.93)	2.1 (0.11)	0.006 (0.012)
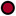	SW2	France (.fr)	22	2.0 (1.3)	2.5 (2.0)	0.59 (1.06)	1.6 (0.13)	0.003 (0.011)

*Note*: Sample size (*n*), Watterson's theta (θ_W_), Tajima's pi (π), Tajima's *D*, heterozygosity – the proportion of heterozygous genotypes and the average population inbreeding coefficients (*F*_IS_) are displayed.

### 3.3 Population structure

**FIGURE 2:**
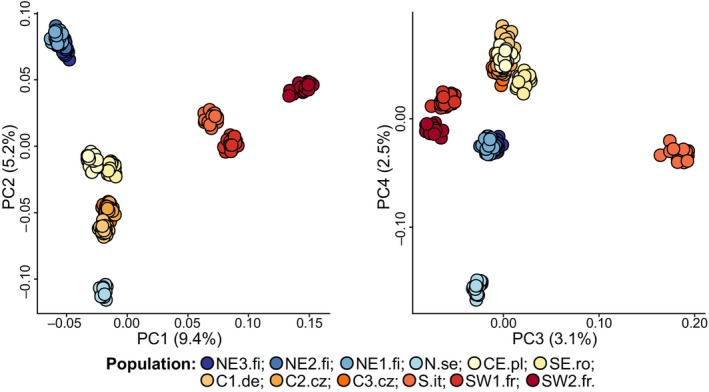
Principal component analysis of 276 *C. glareolus* individuals sampled from 12 populations across Europe using 2476 SNPs based on genotype likelihood. With the percentage of variation explained for each component displayed on the axes, together, the four components explain 20.1% of the variation. Each circle represents an individual, colours correspond to regions and sites. C, Central; E, Eastern; N, North; S, South; W, Western; .fi, Finland; .se, Sweden; .pl, Poland; .cz, Czechia; .de, Germany; it, Italy; .fr, France; .ro, Romania.

PCA of 2476 loci based on genotype likelihood clustered individuals broadly based on geography along the first four principal components (Figure 2), which together explained 20.1% of the genetic variation.

PC1 explained 9.4% of the variation and sorted the populations roughly by geography along a latitudinal gradient (except for N.se, for which individuals had similar values to central populations). PC2 (5.2%) separated populations along a longitudinal axis within regions; in the northern populations, the population west of the Baltic sea (N.se) from the three northern populations east of the Baltic sea (NE1‐3.fi), and within Central Europe, the three geographically close populations (C1.d3, C2‐3.cz) are from the two Eastern European populations (CE.pl and SE.ro). The third and fourth components together explained 5.5% of genetic variation and separated two populations from the other ten: the northern (N.se) population across the Baltic Sea and the southern population on the other side of the Alps (S.it). Similar results were obtained when outlier loci were excluded from the analysis (Figure S2).

**FIGURE 3:**
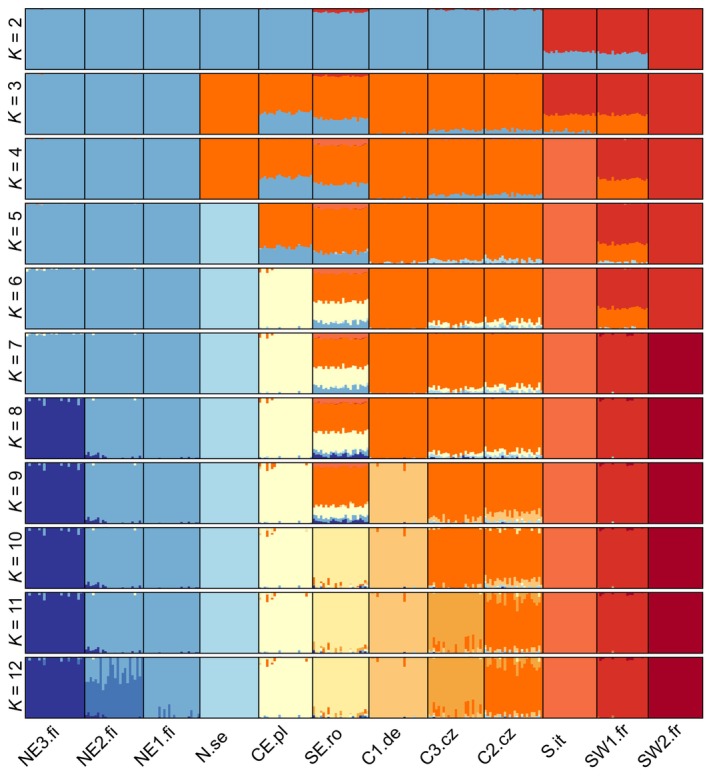
Admixture proportions were estimated using NgsAdmix based on a subset of 2476 SNPs where no individual had missing data. Using different numbers of ancestral populations (*K* = 2–12). Each individual is represented by a column with colours corresponding to the proportions of their ancestry components. Vertical black bars separate putative populations based on sampling location. C, Central; E, Eastern; N, North; S, South; W, Western; .fi, Finland; .se, Sweden; .pl, Poland; .cz, Czechia; .de, Germany; it, Italy; .fr, France; .ro, Romania.

Admixture analysis revealed a clear pattern of genetic clusters (Figure 3). Assuming *K* = 2, the populations in the southern range of the distribution are separated from the other populations. Increasing to *K* = 3 additionally separated the three populations east of the Baltic (NE1‐3.fi), increasing to *K* = 4 separated S.it, and to *K* = 5 the Swedish population (N.se). Assuming *K* = 10 (having the next lowest variance of likelihood), clusters mirrored sampling locations, except for the two pairs of populations with the least geographic distance, the southern Finnish populations forming a single cluster, and the Central populations (C2.cz and C3.cz) with only some degree of admixture suggested, mostly in the Romanian population (SE.ro).

A similar pattern emerges from the pairwise population *F*_ST_ estimates (PPF), which revealed moderate to high levels of differentiation between populations (Table S5). PPF ranged between 0.035 (C1.cz vs. C2.cz) and 0.555 (N.se and SW1.fr) with an average fixation index of 0.269 (SD = 0.12). PPF corresponded well with the geographic proximity of populations (Mantel tests genetic and geographic distance correlation: *r* = .47, *p* = .002), suggesting a strong spatial pattern of isolation by distance. In accordance with previous results, the population from Sweden (east of the Baltic Sea) was more similar to the central European populations than to the North‐Eastern populations. We did not find evidence for IBE using individual climate variables or climate‐based principal components, as there appeared to be no significant association between environmental and genetic distance when controlling for geographic distance (*r* ranging from .12 to .46, all *q* > .05; Table S6).

### 3.4 Candidate loci associated with climate

The univariate approaches identified a total of 975 loci associated with climate variables. To run LFMM, we first determined the appropriate number of latent factors using snmf. The snmf analysis returned the lowest CE value for *K* = 10 (0.519), followed by *K* = 11 (0.520) and *K* = 12 (0.521) (Figure S4). As higher values of *K* resulted in a higher number of outlier loci, we only report outlier loci detected using *K* = 10 as a conservative approach. LFMM identified a total of 497 outlier loci, among which 134 were associated with PC1 and 377 with PC2. Bayenv2 identified a total of 631 outlier loci; of these, 283 loci were associated with PC1 and 354 loci were associated with PC2. Of the outlier loci, 152 were identified by both LFMM and Bayenv2, corresponding to 15.6% overlap between the two methods. The multivariate RDA identified 485 outlier loci associated with the first 2 RDA axes. Among these, 69 loci were also identified by univariate approaches. Thus, 5.0% of loci were identified by both approaches. The RDA without correcting for population structure identified 108 loci as outliers, with only eight loci identified by univariate approaches as well (0.8% overlap with univariate methods) and an overlap of only four loci with the RDA with correction for population structure. Overall, a total of 1392 outlier loci were associated with climate using all methods (Venn Diagrams Figure S3). We considered 213 loci detected by at least two methods as strong candidates.

### 3.5 Annotation of candidate loci and gene ontology

Of the 213 candidate loci, 209 were successfully aligned to the *M. ochrogaster* genome. Eight loci were located in exons, 86 in introns, and 115 in intergenic regions. In total, we identified 74 genes with candidate loci. Some genes contained several loci (Table S7). Several of these genes were associated with functions in lipid metabolism, energy homeostasis, and immunity (Table 2). Among GO terms associated with the genes, six were significantly enriched in our data set, including “regulation of respiratory burst” and “dicarboxylic acid transport” (Table S8).

**TABLE 2:** Candidate genes were detected by at least two genome scan methods and associated with lipid metabolism, energy homeostasis, and immunity.

Locus and method	Gene, putative function and relevance	References
NC_022011.1_19197181	Zinc finger homeobox 3 (Zfhx3)	Balzani et al. (2016)
L1, L2, Ba1	Transcription factor expressed in the suprachiasmatic nucleus with a role in circadian rhythms	
NC_022013.1_77189997	Basic leucine zipper ATF‐like transcription factor 3 (Batf3)	Murphy et al. (2013)
L1, Ba1, Ba2, RDA	Transcription factor involved in the differentiation of T helper cells	
NC_022013.1_78476899	Potassium voltage‐gated channel subfamily H member 1 (Kcnh1)	Zhang et al. (2013)
L2, Ba2	Involved in adipogenic differentiation and production	
NC_022017.1_17053779	ADAM metallopeptidase with thrombospondin type 1 motif 20 (Adamts20)	Silver et al. (2008)
L2, Ba2	Required for melanoblast survival, responsible for coat colour variation	
NC_022018.1_59867563	Neurotrophic receptor tyrosine kinase 2 (Ntrk2)	Xu & Xie (2016), Houtz et al. (2021)
L2, Ba2, RDA	Critical for maintaining energy homeostasis by controlling food intake and body weight	
NC_022024.1_33246556	Insulin‐like growth factor 1 (Igf1)	Baker et al. (1993), Laron (2001)
L2, Ba2	Involved in energy metabolism and mediating growth and development	
NC_022027.1_54997482	Leucine rich repeat containing 8 VRAC subunit C (Lrrc8c)	Tominaga et al. (2004)
L2, RDA	Associated with early stage adipocyte differentiation	
NC_022028.1_49631929	Dynein axonemal heavy chain 8 (Dnah8)	Söhle et al. (2012)
L2, Ba2	Axonemal dynein influencing lipid metabolism, possibly by reg. of inflammatory processes	
NC_022031.1_30606927	BTB domain and CNC homolog 2 (Bach2)	Kuwahara et al. (2016), Yamashita & Kuwahara (2018)
L1, L2, Ba2, RDA	Transcription factor that acts as a broad regulator of the immune homeostasis	
NW_004949096.1_35517029	Biliverdin reductase A (Blvra)	Barañano et al. (2002)
L2, Ba2	Facilitates conversion of biliverdin to bilirubin protecting against cell damage	
NW_004949099.1_1813079	Aprataxin and PNKP‐like factor (Aplf)	Grundy et al. (2012)
L1, Ba1	Involved in double‐strand DNA break repair by promoting non‐homologous end joining	
NW_004949106.1_3445183	Phospholipase C like 1 (Prip)	Oue et al. (2016)
Ba1, RDA	Modulates fat metabolism and regulates non‐shivering thermogenesis in brown adipose tissue	
NW_004949106.1_7957839	Signal transducer and activator of transcription 4 (Stat4)	Kaplan (2005), Kanematsu et al. (2019)
L2, Ba2	Encodes a transcription factor responsible for T‐helper cell development	
NC_022012.1_47630215	Exophilin 5 (Exph5)	McGrath et al. (2012), Yudin et al. (2017)
LPC1, BPC1, RDA	Plays a role in intracellular vesicle trafficking, mutations in this gene lead to skin fragility	
NC_022030.1_4789630	Solute carrier family 2 member 12 (Slc2a12)	Gil‐Iturbe et al. (2019), Stepanov et al. (2017)
BPC1, RDA	Contributes to insulin‐stimulated glucose uptake in skeletal muscle and adipose tissue	
NC_022013.1_70231999	Dual specificity phosphatase 10 (Dusp10)	Zhang et al. (2004)
BPC1, RDA	Regulator of both innate and adaptive immune resp., req. for T‐cell activation and proliferation	
NW_004949164.1_394094	Engulfment and cell motility 1 (Elmo1)	Sarkar et al. (2017)
BPC1, RDA	Defence mechanisms against invading pathogens by inducing inflammatory responses	

*Note*: Locus refers to the location on the *C. ochrogaster* reference genome (Chromosome_base position). Method of outlier detection (L: LFMM, Ba: Bayenv2, RDA: Redundancy Analysis).

### 3.6 Variance partitioning and identification of important climate variables

**FIGURE 4:**
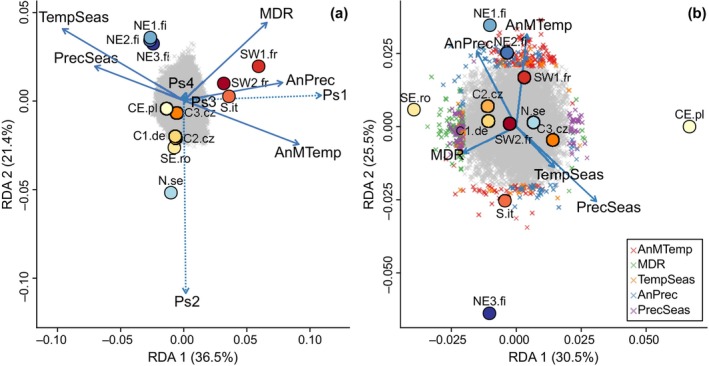
Results of the redundancy analysis with (a) RDA using 20,500 SNPs representing neutral genetic variation (in gray) and five climate variables without correction for population structure. (b) RDA with correction for population structure showing the 485 SNPs identified as outliers by RDA with colour according to the most highly correlated climate variable. Sampling locations are represented by coloured circles. Climate variables are represented by solid blue arrows (AnMTemp: Annual mean temperature, MDR: mean diurnal temperature range, TempSeas: Temperature seasonality, AnPrec: Annual precipitation, PrecSeas: Precipitation seasonality) and control using the four axis of population structure (Ps1‐4) is indicated using dashed blue lines. The arrows' length represents the amount of genetic variation explained by each variable on each axis, and their angle represents the correlation between them. The proportion of total variance explained by each RDA axis is indicated in percent along the axis. Abbreviations for populations according to Figure 1.

We partitioned genetic variation into components of population structure and climate using RDA. Separate RDAs with neutral genetic variation (20,500 SNPs) as the response variable and either population structure or climate as explanatory variables both explained a significant proportion of genetic variation as measured by their adjusted *R* ^2^ (population structure: 66.9%, *p* < .001; climate: 33.1%, *p* = .009). Using RDA, we partitioned this into their exclusive contributions. This showed that population structure when controlling for the effects of climate still explained a significant proportion of genetic variation (38.2%, *p* = .01), while climate when controlling for the effects of population structure did not anymore (4.4%, *p* > .28) (Table S9). Together, population structure and climate explained 71.3% of genetic variation (thus, 28.7% of variation remained unexplained), of which population structure explained 33.0% and climate 16.7%. Thus, the majority of the variation explained was shared between population structure and climate (50.3%) and could not be separated between the two (Table S10). Each climate variable independently explained only a small amount of the total genetic variation in the different subsets of outlier loci and their marginal effects were non‐significant (Figure 4; Table 3).

**TABLE 3:** Amount of genetic variation explained by each climate variable after removing the effect of the other variables including population structure.

Variable	20,500 neutral loci		*p*‐value	485 RDA outlier loci		*p*‐value
	*R* ^2^	Adjusted *R* ^2^		*R* ^2^	Adjusted *R* ^2^	
Annual mean temperature (AnMTemp)	.04	.06	.284	.17	.52	.034*
Temperature seasonality (TempSeas)	.03	.01	.432	.04	.07	.228
Mean diurnal temperature range (MDR)	.04	.06	.267	.12	.35	.058
Annual precipitation (AnPrec)	.04	.05	.290	.08	.20	.128
Precipitation Seasonality (PrecSeas)	.04	.04	.290	.10	.29	.067

*Note*: Results are based on the RDA analysis of climate variables after controlling for population structure for 20,500 neutral loci and 485 outlier loci identified by the RDA analysis. *p < .05

Results from the RDAs performed on the different subsets of outliers vary among the methods used. A significant proportion of variation in the subset of outliers detected by RDA could be attributed exclusively to climate (46.7%, *p* = .023), and the proportion of variation that was shared between both components was reduced to 9.8%. While in the subset of outliers detected by LFMM that were associated with PC1, a total of 63.7% variation could not be attributed to either component, and only 18.6% could be attributed exclusively to climate (Tables S9 and S10).

Most independent climate variables were not significantly associated with genetic variation, and the overall degree of association differed per outlier subset. However, annual mean temperature was significantly associated with genetic variation in the subset of outliers detected by RDA (52.0%, *p* = .034). Within the subset of candidate outliers, annual mean temperature was associated with 23.1% of genetic variation (*p* = .093) followed by annual precipitation (12.8%, *p* > .1). A similar pattern is visible in the results of the variable with the highest influence per outlier marker. Within the subset of candidate loci, annual mean temperature is associated with the highest proportion of outliers (28.6%), followed by annual precipitation (27.7%). Although there is variation between subsets, annual mean temperature is the variable with the highest influence on each marker in most of them, while annual precipitation is the variable with the second highest influence on markers (Figure 4b; Table S11).

### 3.7 Polygenic scores

**FIGURE 5:**
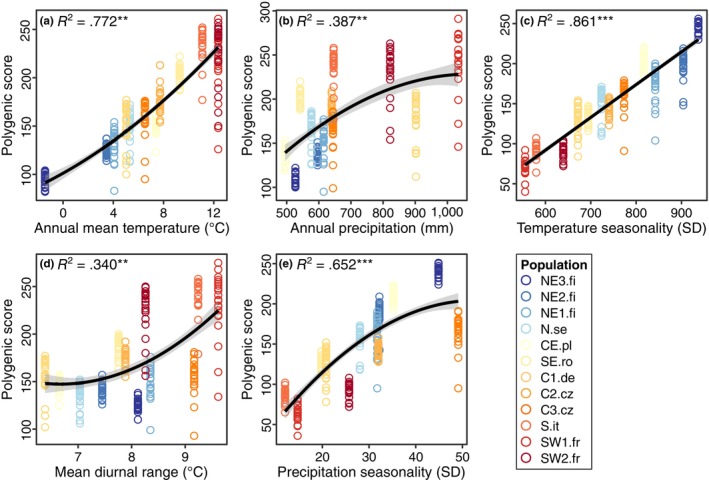
Correlations between individual additive polygenic scores (symbols) based on candidate loci and each of the five corresponding explanatory climate variables determined per sampling site: Annual mean temperature (a), Annual precipitation (b), Temperature seasonality (c), Mean diurnal temperature range (d), and Precipitation seasonality (e). The lines represent the regression line from the model, while the shaded areas represent the 95% confidence interval. The variance explained for the linear (c) or quadratic regression (a, b, d, e) fits is given in the respective upper‐left corner. **p < .01; ***p < .001.

Correlations between additive polygenic scores calculated using candidate loci and the corresponding climate variable were all significant (*p* < .001), and adjusted *R* ^2^ values ranged between .34 (mean diurnal range) and .86 (temperature seasonality) (Figure 5). The correlation between polygenic scores and corresponding climate variables was best represented by quadratic models, except for temperature seasonality, which was best represented by a linear model. The significance and the adjusted *R* ^2^ of the correlations calculated for different subsets of outliers generated similar results but differed slightly across subsets (Table S12).

## 4 DISCUSSION

The aim of the present study was to investigate genome‐wide patterns of adaptive variation associated with climate in European bank vole populations. Searching for potentially adaptive loci in a multivariate framework is a powerful approach, especially because many adaptive traits are likely to be polygenic in nature (Barghi et al., 2020; Wellenreuther & Hansson, 2016). We used ddRAD sequencing and a combination of landscape genomic approaches to uncover climate‐related evolutionary processes in the bank vole. We characterized adaptive genetic variation by combining the results of two univariate and one multivariate GEA methods to detect outlier loci correlated with climate. We identified 74 genes of interest, and functional annotation suggested that energy homeostasis and response to pathogen infection are important targets of climate‐mediated selection in the bank vole. In addition, we have shown that both population history and climate play important roles in explaining genetic differentiation across the bank vole range. Genetic variation among candidate loci was mainly associated with variation in annual mean temperature, highlighting the importance of this climatic variable in bank vole adaptation.

We attempted to distinguish loci with signals of selection that correlated with climate from those loci that exhibit patterns of neutral genetic differentiation. Correcting for the patterns of neutral population structure is an important concern when identifying candidate loci subject to selection. Proper correction can help to avoid the possible spurious detection of candidate loci whose allele frequencies resemble signals of selection but are the result of neutral processes due to the shared history of populations (de Villemereuil et al., 2014). Separating loci under selection from neutral loci becomes increasingly harder when the selective gradients are highly correlated with neutral patterns of population structure (Whitlock & Lotterhos, 2015). In such a situation, the assignment of genetic variation will be confounded between neutral population structure and selective forces. Depending on the correction method used for population structure, this can lead to an increase in false‐negative rates (when variation is excessively assigned to population structure) or to an increase in false‐positive rates (when variation is excessively assigned to selective forces; Forester et al., 2018; Whitlock & Lotterhos, 2015). We attempted to separate genome‐wide patterns of variation into effects of neutral population structure and local adaptation (due to climate). Population structure explained a large part of genomic variation, resulting in a strong pattern of isolation by distance detected using a Mantel test. Similarly, in RDAs population structure still explained a significant amount of variation while controlling for the effects of climate, whereas climate did not when controlling for population structure. Moreover, population structure explained twice as much of the total genetic variation as did climate. These results are not surprising as bank vole populations experience recurring population crashes and effective gene flow between populations is generally low, which results in isolation by distance across both smaller and larger geographic scales (Aars et al., 1998; Gerlach & Musolf, 2000; Guivier et al., 2011; Redeker et al., 2006). Also, bank vole populations have strongly expanded their range since the last glaciation (for review (Kotlik et al., 2022)). Thus, a strong pattern of IBD is to be expected for this species. At the same time, we did not find evidence for IBE using partial Mantel tests with individual climate variables, and climate did not explain significant amounts of variation in an RDA that controlled for population structure. Maybe not unexpectedly, these results suggest that population history is a stronger driver of genetic differentiation in this species than environmental factors. However, it is generally difficult to distinguish a pattern of IBE above the background pattern of IBD when there is a strong correlation between geographic distance and environmental distance (Wang & Bradburst, 2014). The significant correlation between geographic distance and climatic distance (significant for PC1 but not for PC2), together with the large amount of variation that could not be assigned to either population structure or climate may have prevented us from detecting signals of IBE above those of population structure. A large proportion of genetic variation (50.3%) could not be attributed to the exclusive effects of either climate or population structure alone, indicating that a large proportion of genomic variation associated with climate, may be spatially correlated with population structure. The phylogeography of *C. glareolus* is marked by distinct genetic lineages, which resulted from survival within glacial refugia and recolonization of Europe at the end of the last glaciation (Filipi et al., 2015; Horniková et al., 2021; Kotlik et al., 2006; Marková et al., 2020). Post‐glacial expansion from southern refugia may result in clines of neutral allele frequencies coinciding with climate variables related to latitude (Lotterhos & Whitlock, 2014; Rellstab et al., 2015) making it difficult to separate the effects of IBE and IBD from one another. Disentangling these effects would require additional sampling of more populations across the species range, covering gradients within glacial refugia origins. In spite of these limitations, GEA analyses were still able to identify loci putatively under divergent selection, and a RDA on the subset of candidate loci was able to exclusively attribute a proportion of the genetic variation to climate.

There was reasonable overlap in outlier loci detected by the univariate GEA methods LFMM and Bayenv2, which was consistent with results observed in other empirical studies using such methods (Harrison et al., 2017; Prates et al., 2018; Pritchard et al., 2018). While the number of detected outlier loci associated with PC2 was similar between LFMM and Bayenv2 (377 vs. 354), the number of outlier loci associated with PC1 differed markedly between methods (134 vs. 283). This difference may be explained by a stronger collinearity between PC1 and population structure than that of PC2. Surprisingly, the percentage overlap of loci detected by both methods did not differ between PC1 and PC2. Suggesting that LFMM is more conservative than Bayenv2 when the selective gradient and population structure are correlated without affecting the agreement between both methods. The overlap between univariate methods and the RDA with correction for population structure was relatively small, with only 5.1% of the loci detected by the RDA also being detected by LFMM or Bayenv2. GEA methods identify loci under selection after controlling for the effect of neutral drift, and the performance of each method depends on the sampling design and assumed demographic history. This can lead to little overlap between methods (Whitlock & Lotterhos, 2015; de Villemereuil et al., 2014). Population structure and climate were highly collinear in our data set, which makes it harder for GEA methods to separate neutral loci from loci under selection (Whitlock & Lotterhos, 2015). LFMM, Bayenv2, and RDA differed in their ability to separate this from our data. The proportion of variation assigned exclusively to climate ranged from 18.6% to 46.7%, and the proportion shared between population structure and climate ranged from 9.8% to 63.7%. Compared to the subset of neutral loci, the proportion of shared variation decreased, and a larger proportion could be exclusively assigned to climate in the subset of candidate loci. A simulation‐based study that tested the performance of univariate and multivariate GEA methods showed that the performance of these approaches varied depending on the demographic history and strength of selection (Forester et al., 2018) and that RDA may be more robust to our sampling design that does not maximize environmental differentiation. The same study also suggests that combining results from univariate and multivariate approaches may help to increase power and reduce false‐positive rates. As such, our study provides an example of using a conservative approach to outlier detection by combining the results of RDA with GEA methods that use different methods to correct for neutral genetic differentiation in highly structured populations.

Our results suggest that annual mean temperature is an important driver of adaptive genomic variation and thus may be an important selection pressure influencing adaptation in bank vole populations (Tiffin & Ross‐Ibarra, 2014), as reflected by the strong association between temperature and polygenic scores discovered. The latter implies that different alleles are maintained in different thermal environments, suggesting the presence of climate‐mediated selection pressure. Temperature is one of the most important environmental factors affecting physiological processes such as aerobic scope (Pörtner, 2001) and metabolism (Lovegrove, 2003), which in turn affect a variety of life history traits (Simons et al., 2011; Tökölyi et al., 2014). Numerous studies have associated clinal temperature variation and genome scans and found signals of selection in genes related to energy homeostasis and metabolism in endotherms (e.g., Andrew et al., 2018; Fumagalli et al., 2015; Hancock et al., 2011; Harris & Munshi‐South, 2017; Harrison et al., 2017; Lv et al., 2014). This suggests that temperature is one of the most important environmental variables driving local adaptation. Indeed, temperature has been linked to adaptive genetic variation in other small mammals, such as populations of the recently introduced house mouse (*M. musculus*) along a latitudinal cline in eastern North America (Phifer‐Rixey et al., 2018) and populations of the climate‐sensitive American pika (*Ochotona princeps*) along an altitudinal cline (Waterhouse et al., 2018). The distribution and abundance of *C. glareolus* from the Eastern lineage in a contact zone with the Carpathian lineages correlated negatively with July temperature, suggesting that individuals from the Eastern lineage are better adapted to cooler conditions (Tarnowska et al., 2016), supporting temperature as a driver of adaptive genetic variation in the bank vole.

Artificial selection experiments for higher aerobic exercise performance in bank voles resulted in an increase in resting metabolic rate and thus resulted in the development of increased cold tolerance as a side effect (Sadowska et al., 2015; Stawski et al., 2017). This argues for a genetic basis for thermal adaptation in bank voles that may allow individuals under natural conditions to adapt to a colder environment by having more energy available for thermogenesis (Stawski et al., 2017). Although the selection regime increased cold tolerance, it also decreased the ability to thermoregulate at higher temperatures (Grosiak et al., 2020). This suggests that warmer temperatures may also be difficult for small mammals to cope with, as this can easily lead to overheating (Rezende et al., 2004). This in turn could also lead to specific metabolic adaptations in populations in warmer climates due to increased selection pressure. In this study, AMT differed between −1.4°C (NE3.fi) and 12.4°C (S.it) among populations. Thus, bank vole populations in Europe are exposed to quite different environmental temperatures, likely resulting in different energetic requirements and adaptive genetic divergence in metabolic traits throughout the species' range.

Different populations that are exposed to different local climatic conditions not only have different energy requirements but also have to cope with different diets and different pathogen or predator communities. We have identified a number of promising candidate genes that could be considered for future research aimed at linking phenotypic and genotypic variation. The function of these candidate genes could provide insight into the physiological processes that may have undergone selection across climatic gradients. In this context, we have identified a number of candidate genes related to lipid metabolism and the immune system that appear to be subject to temperature‐related selection.

Adipose tissue plays an important role in energy homeostasis and accounts for a large portion of the energy reserves of small mammals (Birsoy et al., 2013; Sethi & Vidal‐Puig, 2007). In particular, brown adipose tissue is important for metabolic heat production through non‐shivering thermogenesis under cold conditions (Cannon & Nedergaard, 2004; Klaus et al., 1988). Two candidate genes associated with lipid metabolism are therefore of particular interest: *PRIP*, which encodes an enzyme that modulates lipid metabolism and serves as a signalling molecule for non‐shivering thermogenesis (Kanematsu et al., 2019; Oue et al., 2016), and *LRRC8C*, which encodes a structural component of the volume‐regulated anion channel in adipocytes and is associated with the early phase of adipocyte differentiation and diet‐induced obesity (Hayashi et al., 2011; Tominaga et al., 2004).

Other candidate genes with functions related to energy homeostasis include *NTRK2*, which encodes the TrkB‐receptor critical for maintaining energy homeostasis by controlling food intake and body weight and is responsible for regulating adaptive thermogenesis (Houtz et al., 2021; Xu & Xie, 2016). Finally, the product of *IGF1* has wide‐ranging effects on metabolism by coordinating protein, carbohydrate, and lipid metabolism in a variety of different cell types (Baker et al., 1993; Laron, 2001). Moreover, several of the candidate genes are associated with obesity in humans, including *DNAH8* (Söhle et al., 2012), *IGF1* (Berryman et al., 2013), *KCNH1* (Vasconcelos et al., 2016), *LRRC8C* (Hayashi et al., 2011), *NTRK2* (Gray et al., 2007), and *PRIP* (Yamawaki et al., 2017) suggesting that they play a role in controlling energy homeostasis and potentially differences in body fat levels across environments.

The results also showed a significant enrichment of genes related to the regulation of the respiratory burst. The respiratory burst plays an important role in the immune system. It is a crucial reaction that occurs in phagocytes to degrade internalized pathogens after phagocytosis (Iles & Forman, 2002). In this context, we have identified a number of candidate genes that play important roles in the immune system. For example, the product of *DUSP10*, which was associated with this significant GO term, plays an important role in regulating both innate and adaptive immune responses through its regulatory influence on the MAPK pathway (Arthur & Ley, 2013; Seternes et al., 2019). Two other candidate genes, *BATF3* and *BACH2*, both encode transcription factors that regulate T‐helper cell function. Interestingly, they also interact with each other to bind to regulatory regions of cytokine gene loci and prevent excessive T‐helper response (Kuwahara et al., 2016; Yamashita & Kuwahara, 2018). Another candidate gene of interest is *STAT4*. This gene encodes a transcription factor responsible for the differentiation of T helper cells (Kaplan, 2005) and is part of the JAK–STAT signalling pathway that controls the immune responses to viral infections (Villarino et al., 2017). JAK–STAT is one of the significantly enriched signalling pathways associated with Puumala hantavirus infection in the bank vole (Rohfritsch et al., 2018). It has also been found to play a role in the immune response to the Sin Nombre hantavirus in deer mice (*Peromyscus maniculatus*) (Schountz et al., 2012, 2014). This suggests that alterations in this gene may be related to Puumala hantavirus infections in the bank vole populations we studied. Similar evidence for differential selection on immune‐related genes has been observed in bank vole populations along environmental gradients at both broad and local scales, using candidate genes (Dubois et al., 2017; Guivier et al., 2014) and other exploratory genome‐wide approaches (Rohfritsch et al., 2018; White et al., 2013). For humans, the diversity of the local pathogenic environment is the predominant driver of local adaptation (Fan et al., 2016), and we may speculate that pathogen environment and pathogen pressure are closely associated with climate variation, with rapid adaptations expected due to climate change. Taken together, the selection signals in the most promising candidate genes suggest that energy balance and the immune system in the bank vole are the most important targets of temperature‐mediated selection.

## 5 CONCLUSIONS

In this study on the genomic adaptation of a small mammal to a pan‐European climate gradient, we have shown that both geographic population structure and climate play important roles in explaining genetic differentiation across the bank vole range. Candidate loci that were detected using GEA while controlling for population structure were most commonly associated with annual mean temperature as the climate variable with the highest explanatory power, highlighting its importance for climate adaptation in the bank vole. We identified 74 genes that showed evidence of climate‐mediated selection and whose functional annotation suggested that energy homeostasis and response to pathogen infection are important targets of selection in the bank vole. We propose to further investigate the functional significance of the identified genes, for example, through common garden experiments and gene expression analysis, as they represent good candidates for local adaptation. Future studies should also look for spatial variation in physiological traits related to energy homeostasis or the immune system to ultimately link genetic variation, organismal physiology, and fitness traits in locally adapted populations.

## AUTHOR CONTRIBUTIONS

**Remco Folkertsma:** Conceptualization (equal); data curation (lead); formal analysis (lead); investigation (lead); methodology (equal); resources (supporting); visualization (lead); writing – original draft (lead); writing – review and editing (equal). **Nathalie Charbonnel:** Resources (supporting); writing – review and editing (supporting). **Heikki Henttonen:** Resources (supporting); writing – review and editing (supporting). **Marta Heroldová:** Resources (supporting). **Otso Huitu:** Resources (supporting); writing – review and editing (supporting). **Petr Kotlík:** Resources (supporting); writing – review and editing (equal). **Emiliano Manzo:** Resources (equal); writing – review and editing (equal). **Johanna L. A. Paijmans:** Methodology (equal); supervision (equal). **Olivier Plantard:** Resources (supporting); writing – review and editing (supporting). **Attila D. Sándor:** Resources (supporting); writing – review and editing (supporting). **Michael Hofreiter:** Conceptualization (equal); methodology (equal); project administration (equal); resources (lead); supervision (equal); visualization (equal); writing – original draft (equal); writing – review and editing (supporting). **Jana A. Eccard:** Conceptualization (equal); project administration (equal); resources (supporting); supervision (equal); writing – original draft (equal); writing – review and editing (lead).

## FUNDING INFORMATION

Czech Science Foundation (grant number 20‐11058S to PK), Deutsche Forschungsgemeinschaft (DFG, German Research Foundation) – Open Access Publications (grant number 491466077 to RF, JAE). Biodiversa+ European Biodiversity Partnership/DFG (grant number 428675001).

## BENEFIT‐SHARING STATEMENT

Benefits Generated: A research collaboration was developed with scientists from the countries providing genetic samples; all collaborators are included as co‐authors; the results of the research have been shared with the provider communities and the broader scientific community (see above); and the research addresses a priority concern, in this case the adaptations of mammals to rising temperatures.

## Supporting information


Figures S1–S4

Tables S1–S12

## Data Availability

The data that support the findings of this study are openly available in Dryad at https://doi.org/10.5061/dryad.1c59zw42p. Raw ddRAD sequencing data for all samples included in this study are available through the National Center for Biotechnology Information's Sequence Read Archive under bioproject number PRJNA1035302. Reviewer sharing link for data on dryad: https://datadryad.org/stash/share/9CmiHRCzjIvAd_Tze9FTG_uHImJb7egkI‐qdLEcv5UM. Reviewer link for sequencing data on NCBI: https://dataview.ncbi.nlm.nih.gov/object/PRJNA1035302?reviewer=p7efvk5uio3grfg7heackn6qf2.
