# Supplementary material for: Genomic signatures of climate adaptation in bank voles
Source: Ecol Evol. 2024 Mar 7;14(3):e10886. doi: 10.1002/ece3.10886 (PMC10918726; doi:10.1002/ece3.10886)
Supplement: Supplementary file 1 — Figures S1–S4 [file ECE3-14-e10886-s002.pdf]

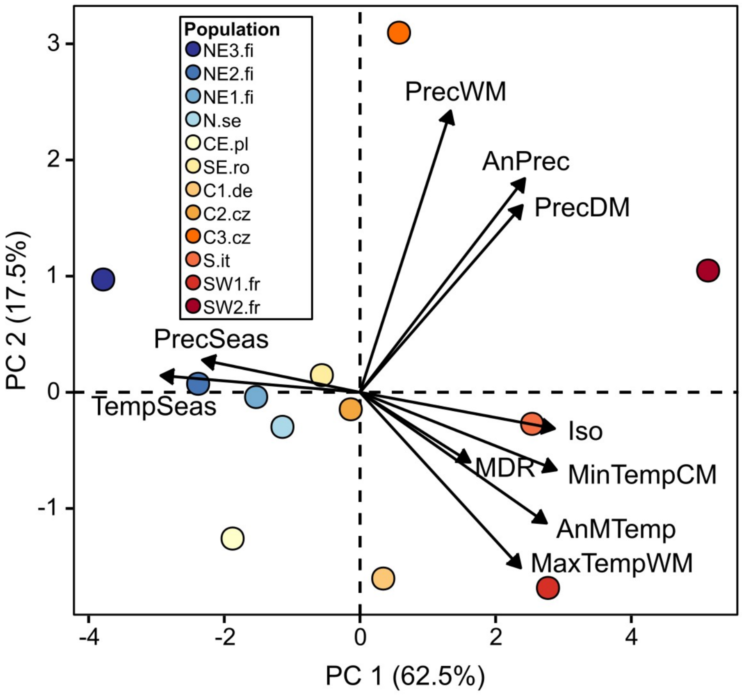

Figure S1. Biplot of the principal component analysis depicting relationships between sampled *C. glareolus* populations (colored circles) and 10 climate variables (depicted by arrows). The proportion of total variance explained by each axis is indicated in percent. Abrreviations for environmental identifiers: AnMTemp: Annual Mean Temperature; MDR:Mean Diurnal Temperature Range; Iso:Isothermality (MDR/TempRange) (* 100); TempSeas: Temperature Seasonality (standard deviation *100); MaxTempWM: Max Temperature of Warmest Month; MinTempCM: Min Temperature of Coldest Month; AnPrec: Annual Precipitation; PrecWM: Precipitation of Wettest Month; PrecDM: Precipitation of Driest Month; PrecSeas: Precipitation Seasonality (Coefficient of Variation).

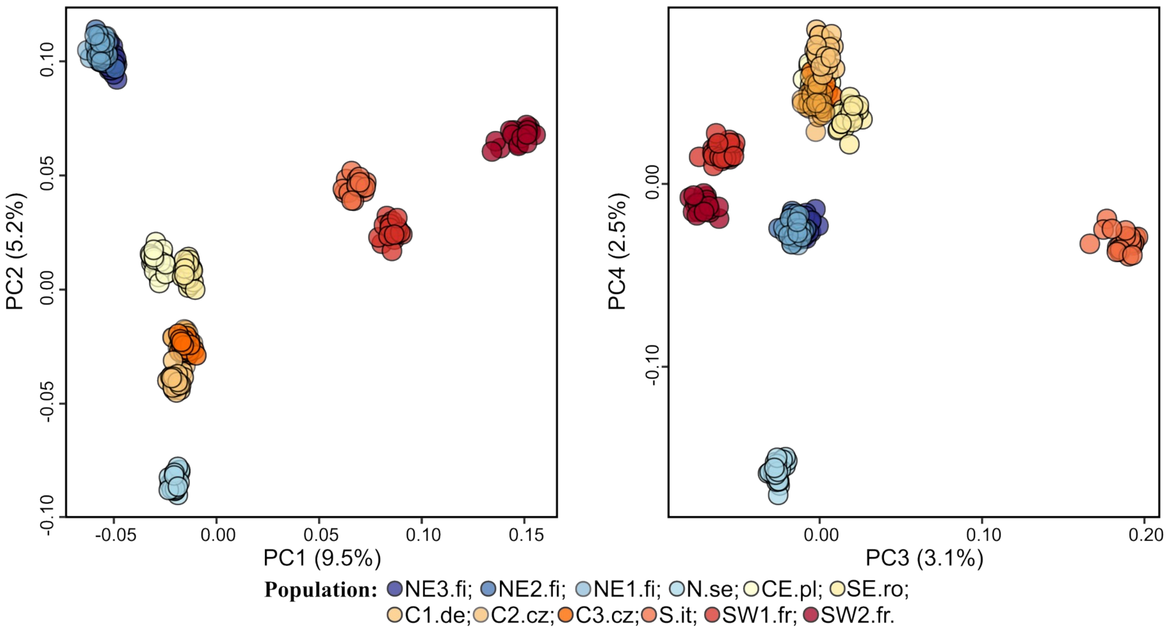

Figure S2. Principal component analysis of 276 *C. glareolus* individuals sampled from 12 populations across Europe. Using the subset of SNPs were no individual has missing data, but also excluding 152 sites with signals of selection (as detected by all outlier methods), resulting in 2,324 SNPs. With percentage of variation explained for each component displayed on the axes, together the four components explain 20.2% of variation. Each circle represents an individual, colours correspond to Regions and sites Abbreviations - C: Central, E: Eastern, N:North, S: South, W: Western, .fi: Finland, .se: Sweden, .pl: Poland, .cz: Czechia, .de: Germany, it: Italy, .fr: France, .ro: Romania

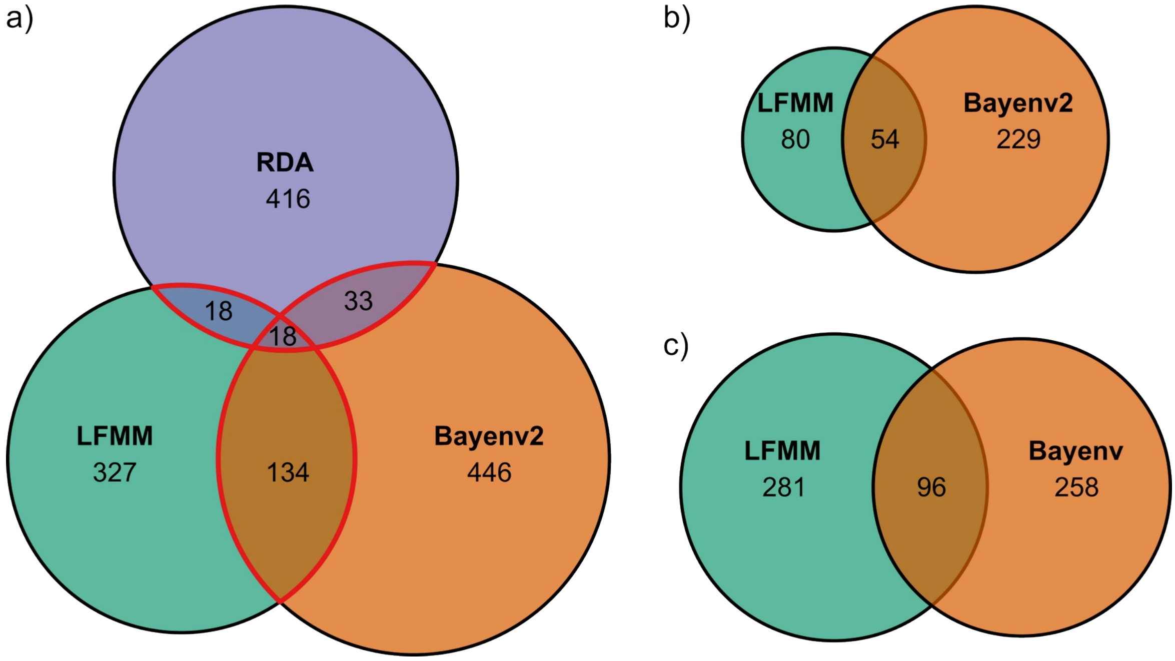

Figure S3. Venn diagrams of outlier loci, size of circles represent the number of outlier loci detected. (a) Comparison of overlap between outliers detected by LFMM, Bayenv2 and RDA. Here we used outliers detected using PC1 and PC2 from LFMM and Bayenv2, outliers that were detected by both PCs are included only once. Candidate loci are indicated in red. (b) Comparison of overlap between outliers detected by LFMM and Bayenv2 using PC1. (c) Comparison of overlap between outliers detected by LFMM and Bayenv2 using PC2.

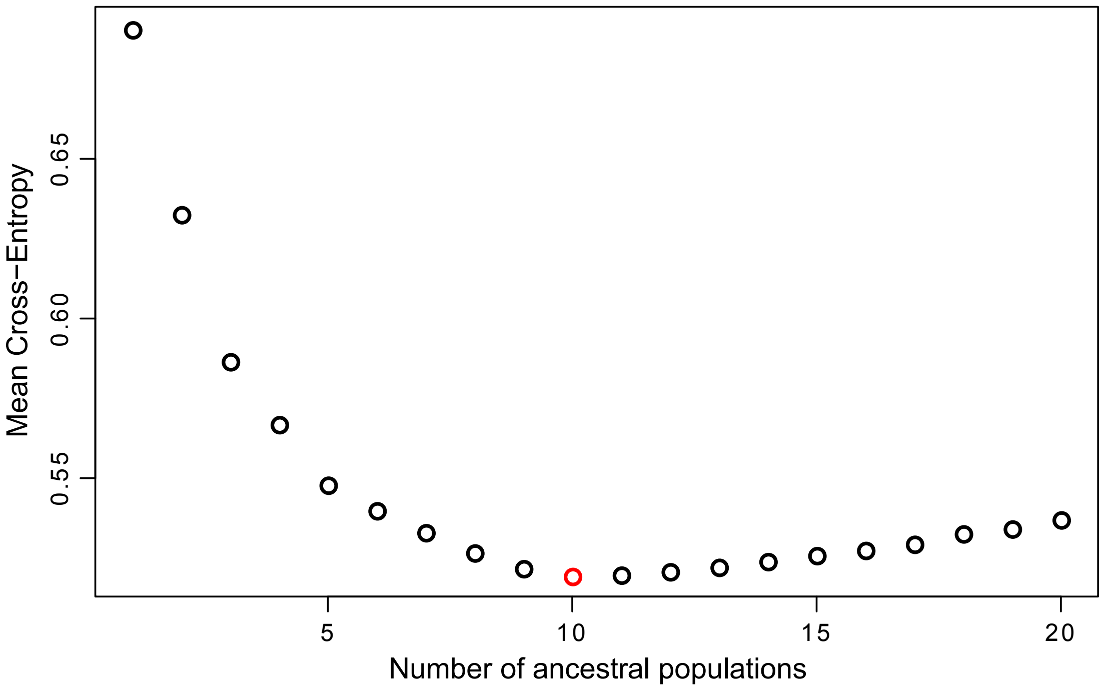

Figure S4. Cross-entropy scores for sNMF runs with numbers of clusters ranging from 1 to 20. The lowest score (*K*=10) is marked in red.
